# Lime-water as a Disinfectant for Linen and Cotton Fabrics

**Published:** 1897-08

**Authors:** 


### LIME-WATER AS A DISINFECTANT FOR LINEN AND COTTON FABRICS.

Beyer has tested different methods employed for the disinfection of bed linen and underclothing. The ordinary methods by boiling are not suited to these articles, as the presence of blood, pus and feces causes an ineradicable stain if a high temperature is used. Soaking the garments in solutions of various soaps for one or two days failed in every instance to kill cholera, typhoid and pyogenic organisms, which were mixed with the feces with which the garments were smeared. In some cases the germs were killed when the solution containing the linen was kept at 50° C. for a few hours. With lime-water the results were much better. Sample garments which were soaked in this solution for twenty-four hours were found to be sterilized. An equally good result was obtained in a hospital where about one-half a cubic meter of soiled linen was soaked in lime-water for forty-eight hours, or for twenty-four hours if the clothing was first rinsed with lime-water, and then placed in a fresh solution. The lime-water does not injure linen or cotton goods, but shrinks woolen to such an extent as to prevent its use —*Fortschrift der Medicine.*

